# Association between osteosarcopenia and coronary artery calcification in asymptomatic individuals

**DOI:** 10.1038/s41598-021-02640-1

**Published:** 2022-04-04

**Authors:** Chul-Hyun Park, Yong-Taek Lee, Kyung Jae Yoon

**Affiliations:** 1 Department of Physical and Rehabilitation Medicine, Kangbuk Samsung Hospital, Sungkyunkwan University School of Medicine, 29 Saemunan-ro, Jongno-gu, Seoul, 03181 Republic of Korea; 2 Department of Health Sciences and Technology, SAIHST, Sungkyunkwan University, Seoul, Republic of Korea

**Keywords:** Cardiology, Endocrine system and metabolic diseases

## Abstract

Osteoporosis and sarcopenia are substantially interrelated with shared cardiovascular risk factors. However, the relationship between osteosarcopenia and coronary artery disease is largely unexplored. We aimed to investigate the association between osteosarcopenia and coronary artery calcification (CAC) scores in asymptomatic adults. A total of 5969 asymptomatic adults without cardiovascular disease who underwent a health examination including estimation of CAC scores by cardiac tomography were analyzed. Osteoporosis was defined as low bone mineral density T-score ≤  − 2.5 standard deviation, and sarcopenia as appendicular skeletal muscle mass < 5.7 kg/m^2^ for women and < 7.0 kg/m^2^ for men, and osteosarcopenia as the copresence of both osteoporosis and sarcopenia. Participants were divided into four groups according to the presence of osteoporosis and/or sarcopenia as control, sarcopenia alone, osteoporosis alone, and osteosarcopenia. Prevalence of CAC was 22.0% in control, 23.6% in sarcopenia alone, 38.5% in osteoporosis alone, and 48.3% in osteosarcopenia group, with the osteosarcopenia group showing the highest (*p* < 0.0001). After adjustments for possible confounders, mean of log (CAC score + 1) in osteosarcopenia group was higher than other three groups (Bonferroni *p* < 0.0001). Using multivariate-adjusted analysis, subjects with osteosarcopenia had the highest risk for having CAC > 0 (odds ratio [OR] 2.868; 95% confidence interval [CI] 1.717–4.790). Furthermore, subjects with osteosarcopenia had a significant risk of moderate-to-extensive CAC (CAC score ≥ 100) (OR 2.709; 95% CI 1.128–6.505). We demonstrated that osteosarcopenia was independently associated with a higher prevalence of subclinical coronary atherosclerosis. Our results suggest osteosarcopenia as a predisposing factor for coronary heart disease.

## Introduction

Osteosarcopenia, a newly identified syndrome characterized by concurrent occurrence of osteoporosis and sarcopenia, is an emerging health challenge in an aging society^1,2^. Muscle and bone are anatomically and functionally linked interrelated musculoskeletal tissues, which provide human locomotion and metabolic storage for calcium in bone and glucose in muscle^1^. Impaired bone health (osteoporosis) commonly occurs when muscle mass or strength is reduced (sarcopenia). The combination of the two is termed osteosarcopenia. Owing to a highly inter-connected nature of bone and muscle, a growing body of research suggests that osteosarcopenia intensifies the risk for fracture, falls, hospitalizations, and further functional decline^3–5^.

Coronary artery disease is another public health threat and a leading cause of morbidity and mortality worldwide^6^. Therefore, early detection of subclinical coronary artery disease is of paramount importance to prevent overt cardiovascular disease (CVD)^7^. Coronary artery calcification (CAC) scoring using computed tomography (CT) is a representative risk marker for coronary atherosclerosis, which reflect the total coronary atherosclerotic burden^8–10^. Furthermore, CAC score can predict future cardiovascular events including mortality and outperforms other risk markers^10^.

Previous studies have revealed that sarcopenia or osteoporosis alone is closely related to CVDs and their risk factors such as hypertension and metabolic syndrome^11–14^. Thus, osteosarcopenia, a combination of osteoporosis and sarcopenia, can be linked to cardiovascular risk. However, there is scanty literature on the relationship of osteoporosis and sarcopenia with subclinical coronary atherosclerosis. To the best of our knowledge, no study has examined the association between osteosarcopenia and subclinical coronary atherosclerosis.

Therefore, this study stratified a cohort of asymptomatic adults by the presence of osteoporosis and/or sarcopenia and evaluated their risk for subclinical coronary atherosclerosis assessed by CAC score.

## Results

### Baseline characteristics

A total of 5969 study participants were divided into four groups according to the presence or absence of sarcopenia and/or osteoporosis as follows: (1) subjects without either abnormality as a control group (n = 4997; 83.7%); (2) subjects with sarcopenia alone (n = 605; 10.1%); (3) subjects with osteoporosis alone (n = 247; 4.1%); and (4) subjects with both abnormalities or osteosarcopenia (n = 120; 2.0%) (Table 1). There were 2696 (45.2%) men and 3273 (54.8%) women. Their mean age and BMI were 49.29 ± 11.22 years and 23.70 ± 3.24 kg/m^2^, respectively (Table 1). Differences in demographic characteristics among the four groups were significant for all variables (all *p* value < 0.0001) with the exception of CRP (*p* = 0.213) and fasting glucose (*P* = 0.230). Proportions of smoking status, alcohol drinking, hypertension, diabetes mellitus, and hyperlipidemia were significantly different among the four groups (all *p* value < 0.0001).

**Table 1:** Baseline characteristics between the groups according to sarcopenia or osteoporosis status.

Variables	Total	Control^a^	Sarcopenia alone	Osteoporosis alone	Osteosarcopenia	*p* value^†^
No. of participants (n)	5969	4997	605	247	120	
Age (year)	49.29 ± 11.22	48.87 ± 10.86	49.73 ± 11.23	55.51 ± 12.02	55.29 ± 11.23	< 0.0001
Male (%)	45.2	44.7	55.9	35.2	30.0	< 0.0001
SBP (mmHg)	111.37 ± 13.33	111.57 ± 13.21	107.83 ± 13.24	115.53 ± 13.08	112.34 ± 15.65	< 0.0001
DBP (mmHg)	71.20 ± 9.69	71.36 ± 9.67	69.54 ± 9.88	72.19 ± 9.45	71.17 ± 9.60	< 0.0001
Height (m)	164.47 ± 8.60	164.87 ± 8.47	164.54 ± 7.91	161.50 ± 10.71	158.94 ± 7.93	< 0.0001
Weight (kg)	64.41 ± 11.85	65.71 ± 11.50	57.69 ± 8.83	64.39 ± 14.89	54.17 ± 8.91	< 0.0001
Waist circumference (cm)	82.22 ± 9.35	82.97 9.14	76.11 ± 8.09	84.48 ± 10.43	77.06 ± 8.55	< 0.0001
BMI (kg/m^2^)	23.70 ± 3.24	24.07 ± 3.12	20.76 ± 2.22	24.46 ± 3.68	21.43 ± 3.07	< 0.0001
ASM (kg)	19.19 ± 4.58	19.61 ± 4.51	16.87 ± 3.84	18.20 ± 5.38	15.30 ± 3.65	< 0.0001
SMI^b^ (kg/m^2^)	6.99 ± 1.06	7.12 ± 1.02	6.23 ± 0.92	6.82 ± 1.14	5.98 ± 0.92	< 0.0001
Current smoker (%)	13.5	13.6	14.9	11.7	7.8	< 0.0001
Heavy drinking (%)	18.1	19.0	15.8	9.6	9.0	< 0.0001
Hypertension (%)	22.4	22.7	15.2	30.4	30.8	< 0.0001
Diabetes mellitus (%)	9.0	9.0	7.8	9.3	14.2	< 0.0001
Hyperlipidemia (%)	26.9	27.1	21.3	37.2	26.7	< 0.0001
AST (IU/L)	21.0 (17.0–26.0)	21.0 (17.0–26.0)	20.0 (17.0–24.0)	23.0 (18.0–29.0)	22.0 (19.0–27.0)	< 0.0001
ALT (IU/L)	19.0 (14.0–28.0)	19.0 (14.0–28.0)	17.0 (13.0–24.0)	22.0 (15.0–32.0)	19.0 (14.0–27.0)	< 0.0001
CRP (mg/dL)	0.05 (0.02–0.10)	0.05 (0.02–0.10)	0.04 (0.02–0.09)	0.05 (0.02–0.10)	0.04 (0.02–0.09)	0.213
Albumin (g/dL)	4.64 ± 0.26	4.63 ± 0.26	4.68 ± 0.25	4.63 ± 0.27	4.63 ± 0.22	< 0.0001
Calcium (mg/dL)	9.47 ± 0.34	9.46 ± 0.33	9.52 ± 0.33	9.51 ± 0.33	9.50 ± 0.32	< 0.0001
Phosphorus (mg/dL)	3.63 ± 0.46	3.62 ± 0.46	3.60 ± 0.48	3.66 ± 0.45	3.82 ± 0.45	< 0.0001
Total cholesterol (mg/dL)	195.26 ± 36.80	195.03 ± 36.86	193.31 ± 36.12	202.43 ± 35.69	199.90 ± 38.32	< 0.0001
Triglycerides (mg/dL)	95.0 (68.0–138.0)	96.0 (68.0–141.0)	85.0 (63.0–126.0)	101.0 (74.5–148.0)	89.0 (60.0–117.8)	< 0.0001
HDL-C (mg/dL)	59.48 ± 15.95	59.11 ± 15.89	61.28 ± 16.06	59.34 ± 15.07	63.11 ± 17.82	< 0.0001
LDL-C (mg/dL)	126.65 ± 34.83	126.76 ± 34.82	122.48 ± 34.48	133.28 ± 34.82	129.65 ± 36.02	< 0.0001
Fasting glucose (mg/dL)	97.41 ± 16.99	97.41 ± 16.38	96.51 ± 19.28	98.94 ± 16.49	98.60 ± 27.46	0.230
Fasting insulin (IU/L)	6.47 ± 4.14	6.64 ± 4.18	5.11 ± 3.17	6.99 ± 4.80	5.28 ± 3.69	< 0.0001
HbA1c (%)	5.69 ± 0.61	5.69 ± 0.60	5.65 ± 0.66	5.76 ± 0.56	5.80 ± 0.87	< 0.0001
HOMA-IR	1.31 (0.85–1.97)	1.35 (0.88–2.02)	1.06 (0.74–1.48)	1.30 (0.88–2.10)	1.04 (0.93)	< 0.0001

Values are expressed as mean ± standard deviation, median (interquartile range) or percentage.

SBP, systolic blood pressure; DBP, diastolic blood pressure; BMI, Body Mass Index; ASM, appendicular skeletal muscle mass; AST, aspartate aminotransferase; ALT, alanine aminotransferase; CRP, C-reactive protein; HDL-C, high-density lipoprotein cholesterol; LDL-C, low-density lipoprotein cholesterol; HbA1c, glycated hemoglobin; HOMA-IR, homeostasis model assessment of insulin resistance.

^†^*p* value for group difference analyzed by one-way ANOVA in continuous variables or by Chi-square test in categorical variables.

^a^Non-sarcopenic/non-osteoporotic group.

^b^SMI (Skeletal muscle mass index) (kg/m^2^) was calculated as ASM (kg)/height (m)^2^.

### Comparison of coronary artery calcification between Groups according to sarcopenia or osteoporosis status

Of the total population, 23.3% had coronary artery calcification (CAC score > 0). The prevalence of coronary artery calcification was 22.0% in the control group, 23.6% in the sarcopenia alone group, 38.5% in the osteoporosis alone group, and 48.3% in the osteosarcopenia group, with the osteosarcopenia group showing the highest prevalence (*p* < 0.0001) (Table 2). The prevalence of moderate-to-extensive CAC (CAC score ≥ 100) was 6.5% in normal, 6.9% in sarcopenia alone, 10.9% in osteoporosis alone, and 15.0% in osteosarcopenia, with the osteosarcopenia group showing the highest (*p* < 0.0001).

**Table 2:** Prevalence of coronary artery calcification among the four groups according to sarcopenia or osteoporosis status.

Variables	Control^a^	Sarcopenia alone	Osteoporosis alone	Osteosarcopenia	*p* value^†^
Number of subjects	4997	605	247	120	
Prevalence of subjects with CAC score = 0 (%)	78.0	76.4	61.5	51.7	< 0.0001
Prevalence of subjects with CAC score > 0 (%)	22.0	23.6	38.5	48.3	< 0.0001
Prevalence of subjects with CAC score > 100 (%)	6.5	6.9	10.9	15.0	< 0.0001

CAC, coronary artery calcification.

^a^Non-sarcopenic and non-osteoporotic group.

^†^*p* value for group difference analyzed by Chi-square test.

**Figure 1:**
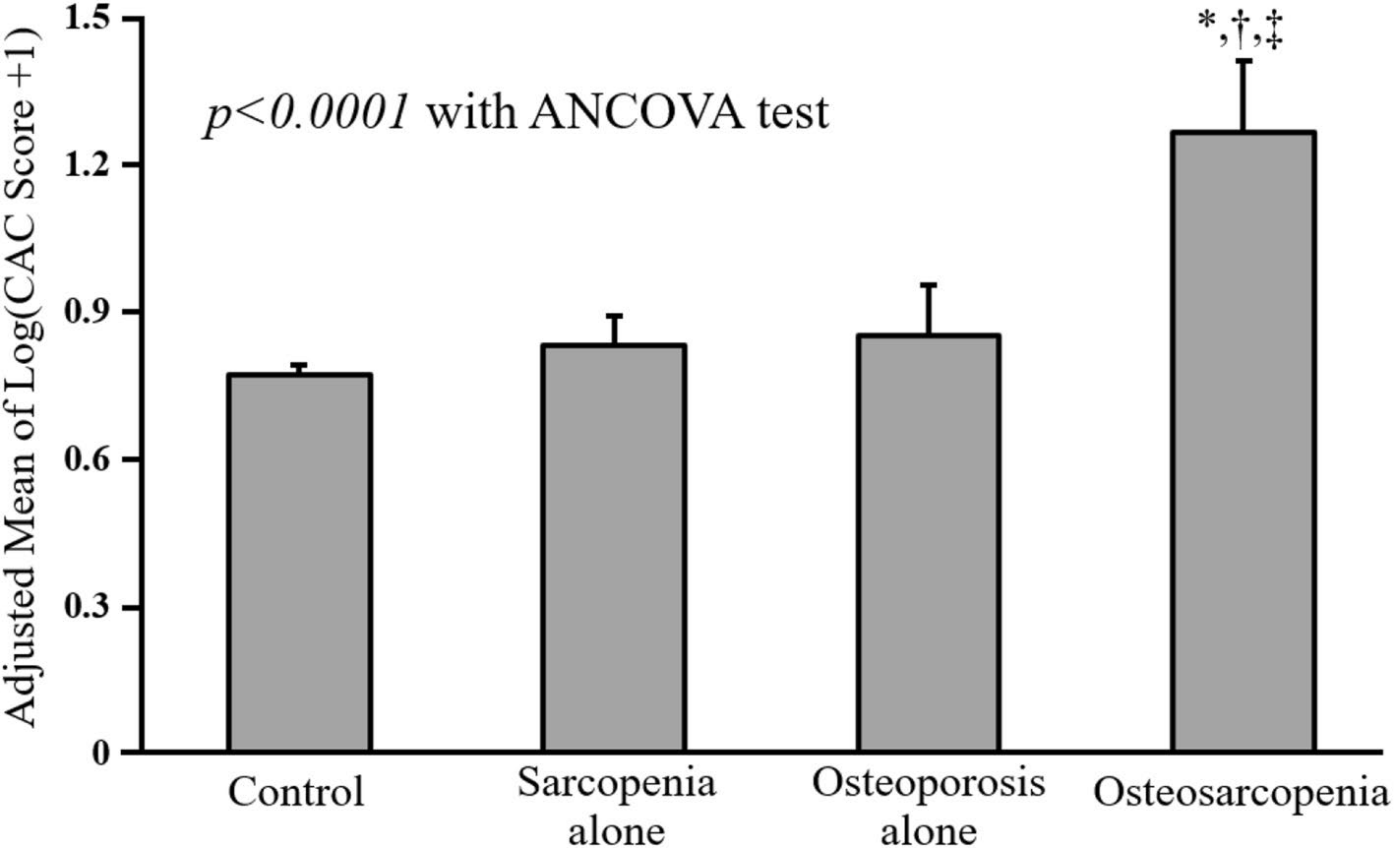
Comparison of Adjusted means of coronary artery calcification score between groups according to sarcopenia or osteoporosis status. Adjusted means of CAC score were estimated from ANCOVA after adjustments for age, sex, screening center, triglycerides, BMI, history of hypertension, CRP, HOMA-IR, smoking status, and alcohol intake. *Adjusted *p* < 0.0001 versus control in Bonferroni post-hoc analysis. ^†^Adjusted *p* < 0.0001 versus sarcopenia alone in Bonferroni post-hoc analysis. ^‡^Adjusted *p* < 0.0001 versus osteoporosis alone in Bonferroni post-hoc analysis. CAC, coronary artery calcification; BMI, body mass index; CRP, c-reactive protein; HOMA-IR, homeostatic model assessment of insulin resistance.

The mean log (CAC score + 1) was 0.841. After adjustments for age, sex, screening center, triglycerides, BMI, history of hypertension, CRP, HOMA-IR, smoking status, and alcohol intake, the adjusted mean of log (CAC score + 1) in osteosarcopenia group was higher than other three groups (all *p* value < 0.0001, Bonferroni post-hoc analysis) (Fig. 1).

### Risk of coronary artery calcification in subjects with osteosarcopenia

A multivariable logistic regression analysis was performed to determine ORs for prevalence of CAC (CAC score > 0) according to sarcopenia and osteoporosis status (Table 3). Subjects with osteosarcopenia had the highest risk for CAC (Model 4, adjusted OR 2.868; 95% CI 1.717–4.790) after adjusting for confounding variables. Although sarcopenia alone was not associated with CAC (adjusted OR 0.995; 95% CI 0.746–1.327), subjects with osteoporosis alone had an increased risk for CAC (adjusted OR 1.610; 95% CI 1.129–2.296).

**Table 3:** Multivariable-adjusted odds ratios (95% CI) for prevalence of coronary artery calcification in sarcopenia, osteoporosis, and osteosarcopenia.

	Control^a^	Sarcopenia alone	Osteoporosis alone	Osteosarcopenia
**CAC score > 0 (%)**				
Model 1	1.00 (Ref)	1.100 (0.902–1.343)	2.223 (1.705–2.896)	3.326 (2.310–4.787)
Model 2	1.00 (Ref)	0.850 (0.666–1.085)	1.742 (1.300–2.334)	3.151 (2.052–4.840)
Model 3	1.00 (Ref)	0.975 (0.737–1.292)	1.731 (1.239–2.418)	3.114 (1.906–5.088)
Model 4	1.00 (Ref)	0.995 (0.746–1.327)	1.610 (1.129–2.296)	2.868 (1.717–4.790)
**CAC score ≥ 100 (%)**				
Model 1	1.00 (Ref)	1.091 (0.780–1.526)	2.132 (1.394–3.259)	3.484 (2.037–5.959)
Model 2	1.00 (Ref)	0.702 (0.463–1.063)	1.612 (1.008–2.687)	3.030 (1.540–5.962)
Model 3	1.00 (Ref)	0.851 (0.526–1.375)	1.515 (0.841–2.728)	3.203 (1.414–7.256)
Model 4	1.00 (Ref)	0.882 (0.536–1.454)	1.595 (0.871–2.920)	2.709 (1.128–6.505)

CI, confidence interval; CAC, coronary artery calcification; OR, odds ratios; BMI, body mass index; CRP, C-reactive protein; HOMA-IR, homeostasis model assessment of insulin resistance.

Model 1: Crude model.

Model 2: Adjusted for age, sex, screening center, triglycerides.

Model 3: Model 2 + BMI, history of hypertension, CRP, HOMA-IR.

Model 4: Model 3 + smoking status, alcohol intake.

^a^Non-sarcopenic and non-osteoporotic group.

In multivariable logistic analyses with moderate-to-extensive CAC (CAC score ≥ 100) as a dependent variable, osteosarcopenia had a high risk for moderate-to-extensive CAC (Model 4, adjusted OR 2.709; 95% CI 1.128–6.505). While subjects with osteoporosis alone had an increased risk for moderate-to-extensive CAC in crude analysis (Model 1, OR 2.132; 95% CI 1.394–3.259), the risk of CAC score ≥ 100 was not statistically significant after adjusting for covariates (Model 4, adjusted OR 1.595; 95% CI 0.871–2.920). Subjects with sarcopenia alone did not show significantly increased risk of CAC score ≥ 100 after adjusting for covariates (adjusted OR 0.882; 95% CI 0.536–1.454).

When similar analyses were conducted with CAC score as a continuous variable, the CAC score showed positive correlation with osteosarcopenia even after adjusting for confounding variables (Model 4, adjusted coefficients: 1.436; 95% CI 1.061–1.944) (Table 4). Subjects with either sarcopenia alone or osteoporosis alone had no significant association with CAC score after adjustments for covariates.

**Table 4:** Multivariable-adjusted coefficients (95% CI) for CAC Score in sarcopenia, osteoporosis, and osteosarcopenia.

	Control^a^	Sarcopenia alone	Osteoporosis alone	Osteosarcopenia
Model 1	1.00 (Ref)	1.091 (0.780–1.526)	2.132 (1.394–3.259)	3.484 (2.037–5.959)
Model 2	1.00 (Ref)	1.005 (0.885–1.142)	1.244 (1.025–1.509)	1.942 (1.477–2.553)
Model 3	1.00 (Ref)	1.070 (0.937–1.221)	1.146 (0.943–1.392)	1.880 (1.432–2.470)
Model 4	1.00 (Ref)	1.016 (0.884–1.166)	1.166 (0.940–1.446)	1.436 (1.061–1.944)

CI, confidence interval; CAC, coronary artery calcification; OR, odds ratios; BMI, body mass index; CRP, C-reactive protein; HOMA-IR, homeostasis model assessment of insulin resistance.

Estimated from multivariate general linear models used with natural log (CAC score + 1) as the outcome.

Model 1: Crude model.

Model 2: Adjusted for age, sex, screening center, triglycerides.

Model 3: Model 2 + BMI, history of hypertension, CRP, HOMA-IR.

Model 4: Model 3 + smoking status, alcohol intake.

^a^Non-sarcopenic and non-osteoporotic group.

### Subgroup analyses by clinically relevant factors

The association between osteosarcopenia and the presence of CAC was next examined for clinically relevant subgroups including age, sex, and other regarding factors (Table 5). The association between osteosarcopenia and CAC was similar between women and men with no significant interactions (*p* for interaction = 0.956). The association of osteosarcopenia and CAC was stronger in younger participants (< 60 years) than in older participants (≥ 60 years) (*p* for interaction < 0.0001). We conducted a further analysis in subgroups divided by sex and age (Table 6). Younger women had a strong association between osteosarcopenia and CAC (adjusted OR = 3.711; 95% CI 1.619–8.507), whereas older women had insignificant association between osteosarcopenia and CAC (adjusted OR 0.882; 95% CI 0.536–1.454) (*p* for interaction < 0.0001). A strong association between osteosarcopenia and CAC was present in young men (adjusted OR 3.303; 95% CI 1.272–8.579) although there was no significant association among older men (adjusted OR 0.882; 95% CI 0.536–1.454) (*p* for interaction < 0.0001).

**Table 5:** Subgroup analysis for prevalence of coronary artery calcification in sarcopenia, osteoporosis, and osteosarcopenia.

Subgroups	Control^a^	Sarcopenia alone	Osteoporosis alone	Osteosarcopenia	*p* for interaction
**Sex**					
Female (n = 3273)	1.00 (Ref)	0.967 (0.531–1.763)	1.423 (0.873–2.320)	2.588 (1.341–4.991)	0.956
Male (n = 2696)	1.00 (Ref)	1.020 (0.734–1.418)	1.711 (1.000–2.927)	3.213 (1.346–7.669)	
**Age**					
< 60 years (n = 4805)	1.00 (Ref)	1.036 (0.740–1.449)	1.653 (1.038–2.631)	4.034 (2.174–7.484)	< 0.0001
≥ 60 years (n = 1164)	1.00 (Ref)	1.080 (0.658–1.775)	1.158 (0.664–2.020)	2.442 (1.078–5.533)	
**Smoking status**					
Current smoker (n = 751)	1.00 (Ref)	1.317 (0.714–2.428)	2.683 (1.079–6.669)	1.959 (0.398–9.648)	0.720
Non- or Ex-smoker (n = 5218)	1.00 (Ref)	0.927 (0.668–1.286)	1.465 (0.995–2.158)	2.983 (1.735–5.128)	
**Alcohol intake**					
< 20 g/d (n = 4430)	1.00 (Ref)	0.933 (0.669–1.130)	1.528 (1.046–2.231)	3.245 (1.896–5.553)	0.397
≥ 20 g/d (n = 1539)	1.00 (Ref)	1.236 (0.682–2.239)	2.739 (0.929–8.073)	0.930 (0.185–1.241)	
**HOMA-IR**					
< 2.5 (n = 4943)	1.00 (Ref)	1.006 (0.738–1.371)	1.596 (1.063–2.396)	2.899 (1.672–5.028)	0.153
≥ 2.5 (n = 1026)	1.00 (Ref)	0.950 (0.411–2.196)	1.799 (0.834–3.881)	2.910 (0.615–13.769)	
**BMI (kg/m**^**2**^)					
< 23.0 (n = 2606)	1.00 (Ref)	0.913 (0.626–1.331)	1.926 (0.957–3.877)	1.952 (1.008–3.782)	0.002
≥ 23.0 (n = 3363)	1.00 (Ref)	1.202 (0.700–2.064)	1.524 (1.009–2.302)	8.692 (3.154–23.954)	

Multivariable-adjusted odds ratios (95% CI) were estimated after adjustments for age, sex, screening center, triglycerides, BMI, history of hypertension, CRP, HOMA-IR, smoking status, and alcohol intake.

CI, confidence interval; CAC, coronary artery calcification; OR, odds ratios; BMI, body mass index; CRP, C-reactive protein; HOMA-IR, homeostasis model assessment of insulin resistance.

^a^Control = non-sarcopenic and non-osteoporotic.

**Table 6:** Subgroup analysis of sex and age (4 categories) for prevalence of coronary artery calcification in sarcopenia, osteoporosis, and osteosarcopenia.

Subgroups	Control^a^	Sarcopenia alone	Osteoporosis alone	Osteosarcopenia	*p* for interaction
**Women (n = 3273)**					
< 60 years (n = 2670)	1.00 (Ref)	1.076 (0.509–2.275)	2.266 (1.003–5.120)	3.711 (1.619–8.507)	< 0.0001
≥ 60 years (n = 603)	1.00 (Ref)	0.708 (0.261–1.919)	1.037 (0.566–1.901)	1.670 (0.600–4.648)	
**Men (n = 2696)**					
< 60 years (n = 2135)	1.00 (Ref)	1.029 (0.703–1.506)	1.197 (0.693–2.068)	3.303 (1.272–8.579)	< 0.0001
≥ 60 years (n = 561)	1.00 (Ref)	1.215 (0.662–2.229)	2.358 (0.253–22.009)	3.434 (0.681–17.325)	

Multivariable-adjusted odds ratios (95% CI) were estimated after adjustments for age, sex, screening center, triglycerides, BMI, history of hypertension, CRP, HOMA-IR, smoking status, and alcohol intake.

CI, confidence interval; CAC, coronary artery calcification; OR, odds ratios; BMI, body mass index; CRP, C-reactive protein; HOMA-IR, homeostasis model assessment of insulin resistance.

^a^Control = non-sarcopenic and non-osteoporotic.

Overweight participants (BMI ≥ 23.0) had a stronger association between osteosarcopenia and CAC than participants with BMI < 23.0 (*p* for interaction < 0.0001) (Table 5). The association between CAC and osteosarcopenia was similar across participant subgroups, showing no significant interaction between smoking status (current smoker vs. never or ex-smoker), alcohol intake (< 20 g/day vs. ≥ 20 g/day), and HOMA-IR (< 2.5 vs. ≥ 2.5).

## Discussion

In this study, we demonstrated that osteosarcopenia was independently associated with an increased risk of subclinical coronary artery disease and associated with moderate-to-extensive CAC. These associations remained significant even after adjusting for possible confounding variables. They were consistently observed in various subgroups. To the best of our knowledge, this is a first study to report osteosarcopenia as a risk factor for early coronary atherosclerosis as measured by CAC score in apparently healthy adults. Furthermore, we found a considerably strong association between osteosarcopenia and CAC in younger (< 60 years) and overweight subjects.

As demonstrated by the present study, subjects with osteosarcopenia had a higher risk of subclinical coronary artery disease than those with sarcopenia or osteoporosis alone. Although most studies on osteosarcopenia are based on epidemiological data^3,15,16^, there is substantial evidence of pathophysiological pathways supporting an intimate connection between osteoporosis and sarcopenia. Growth hormone and insulin-like growth factor (GH/IGF-1) play a principal role in the development of osteosarcopenia^17,18^. GH/IGF-1 promotes osteoblast proliferation and inhibits osteoclast activity^19^. Furthermore, GH affects muscle fiber size, type, and cell proliferation by IGF-1 activity^18^. Another key mechanism is ‘inflammaging’ which describes that muscle and bone are likely connected^20^. This term indicates a status of chronic low-grade inflammation with aging that presents an increased production of pro-inflammatory cytokines^21^. Pro-inflammatory cytokines are highly linked to the development of sarcopenia through the ubiquitin-protease pathway, resulting in acceleration of bone resorption^22,23^. Therefore, sarcopenia and osteoporosis are intricately connected, affecting each other through chemical and molecular pathways. At present, a study on Iranian elderly has revealed a significant association of osteosarcopenia with cardiovascular risk factors such as BMI and high fat mass^24^. However, to the best of our knowledge, no study has reported the relationship between osteosarcopenia and coronary heart disease. We firstly report an increased risk of coronary atherosclerotic burden in subjects with osteosarcopenia.

Our results are in line with previous studies which demonstrated a close relationship between osteoporosis and coronary atherosclerotic plaque^25,26^. A retrospective study of 246 Taiwan patients has noted that CAC is associated with osteoporosis^25^. The Copenhagen General Population study has shown an inverse relation between BMD and CAC in both men and postmenopausal women^26^. Furthermore, a cohort study of 5590 subjects without known coronary artery disease has reported that low BMD level is an independent predictor for the presence of CAC and mortality^27^. Possible mechanism for the relationship between osteoporosis and CAC might involve a shift in mineralization from skeletal bone to coronary arteries^28^. Thus, an increased bone calcium resorption can mediate the accumulation of coronary artery calcium^29^. Furthermore, vascular calcification and bone loss can both be affected by aging-related changes in bone metabolism and integrity of vasculature^30^. As shown in this study, osteoporosis alone was significantly associated with the presence of CAC even after adjusting for other covariates.

This study showed an insignificant association between sarcopenia alone and CAC. However, the coexistence of osteoporosis and sarcopenia (osteosarcopenia) showed a stronger risk of CAC than osteoporosis alone, further increasing the risk for moderate-to-extensive CAC (CAC ≥ 100). There are several explanations. First, considering muscle mass comprises approximately 85% of glucose consumption in the body, metabolic implication of muscle on cardiovascular condition has been noted^31^. Previous studies have described that insulin resistance can mediate the association between muscle mass and CVD risk^32^. Increased insulin resistance can induce autophagy activity by suppressing the target of rapamycin pathway, which resulted in muscle loss^33^. Thus, dysfunction in musculature as a primary site of insulin-mediated glucose disposal in whole body can potentially affect a CVD risk. Second, low muscle mass can affect an arterial stiffness estimated using a brachial-ankle pulse wave velocity. Recently, the Wakayama study of 1046 elderly without history of CVD has indicated that low muscle mass shows an increased arterial stiffness and that a combination of low muscle mass and strength can lead to greater arterial stiffness^34^. Because an increased arterial stiffness is strongly associated with coronary artery disease and CVD events^35^, individuals with loss in muscle mass combined with osteoporosis could accelerate coronary artery atherosclerosis compared to individuals with osteoporosis alone. Therefore, although sarcopenia itself is insufficient to affect the development of CAC, sarcopenia could be an intermediate or indirect predisposing factor affecting coronary artery condition rather than an independent risk factor. We presented that osteoporosis alone was independently associated with an increased risk of CAC, although was not associated with moderate-to-extensive CAC (> 100). Therefore, the impact of osteosarcopenia on the development CAC could be mainly caused by osteoporosis than sarcopenia. Therefore, sarcopenia may potentiate adverse effects on coronary artery atherosclerosis only in the condition of osteoporosis.

In this study, younger (< 60 years) subjects showed a strong association of osteosarcopenia with coronary atherosclerosis than older (≥ 60) subjects. Interestingly, in elderly subjects (≥ 60 years), sarcopenia, osteoporosis, and osteosarcopenia status were not associated with the presence of CAC in both men and women. This finding is in line with a previous cohort study showing that the association between obesity and coronary heart disease was significant only in younger subjects, but not in the elderly aged over 60 years^36^. Furthermore, the association between obesity and coronary heart disease declined with aging. Other previous studies also reported a higher association between metabolic parameters and mortality risk in younger subjects than older adults^37,38^. Stevens et al. have shown that obesity in younger-aged subjects has an increased association with all-cause mortality than that in older ones^38^. A study from Canadian National Health and Nutrition Survey has demonstrated that younger adults having all five components of metabolic syndromes have a stronger association with mortality risk than older men^37^. Therefore, these similar patterns of the age-dependent attenuation in the associations may partially explain the insignificant relationship between osteosarcopenia and CAC in the elderly. Because osteoporosis and sarcopenia are considered as metabolic conditions, aging may have a similar influence on the metabolic effect of osteosarcopenia on coronary artery disease. Although osteosarcopenia is an age-related condition, it can occur in younger and middle-aged adults, showing high correlations with inflammation parameters. Recently, we have reported that there is a significant association between high-sensitivity CRP (hs CRP) and sarcopenic obesity, with stronger association in young adults than in the elderly^39^. CRP as a representative inflammation marker is known to be closely connected to the extent and progression of coronary atherosclerosis^40^. Because sarcopenia is a well-known low-grade inflammation disease^41^, a subclinical inflammation can be an important mediator for the relation between osteosarcopenia and CAC. Nevertheless, it is not clear why the association of osteosarcopenia with the CAC was not significant in the elderly. Future prospective research is needed to focus on the age difference between osteosarcopenia and incident CAC.

We demonstrated that subjects with overweight/obese (BMI ≥ 23.0) had a considerably stronger association of osteosarcopenia with CAC than subject with normal/underweight (BMI < 23.0). Osteosarcopenia combined with overweight/obese is a newly suggested condition called osteosarcopenic obesity (OSO) that simultaneously has abnormalities of bone, muscle, and adiposity^42^. A recent study on Chinese adults has shown that subjects with OSO have a higher risk for hypertension than those with two abnormalities of bone, muscle, and adiposity^43^. Furthermore, OSO is significantly associated with low vitamin D level^44^. Because low vitamin D level is known to be correlated with increased CAC score^45^, subjects with osteosarcopenia combined with overweight/obese related to low vitamin D can be at more risk for increased CAC than those with normal/underweight.

### Limitations

This study has several limitations. First, the population mostly comprised middle-aged metropolitan Korean adults, which limits generalizability of our findings. However, to overcome this, we added various subgroup analyses stratified by age of 60 years, sex, smoking status, alcohol intake, BMI, and HOMA-IR. Furthermore, data were collected from two health screening centers from different cities in Korea to reduce selection bias. Second, this study could not consider muscular function for defining sarcopenia. Generally, after muscle mass starts to decline, physical strength and performance status also deteriorate for those over 50 years^46^. Due to the relatively younger age of subjects, the present study considered only the quantity of muscle mass for defining sarcopenia. Moreover, many previous studies were reported with defining sarcopenia based on the low muscle mass alone^44,47,48^. Third, this was a cross-sectional design which prevented our accessing causal relationships. However, we tried to investigate the direct relationship between osteosarcopenia and coronary atherosclerosis independent of many possible confounding variables. A further prospective study building on the second-wave follow-up data of this cohort is needed to explore the progression of CAC in individuals with osteosarcopenia.

## Conclusions

In this large sample of apparently healthy adults, an increased risk of prevalent subclinical coronary atherosclerosis was found for those with osteosarcopenia, even if they were comparatively middle-aged. In addition, osteosarcopenia was associated with advanced coronary artery disease. These associations were higher in younger (< 60 years) and overweight/obese subjects than in older and normal/underweight subjects. This study suggests a strong connection between osteosarcopenia and the development of coronary atherosclerosis. Prospective randomized studies are needed to show that treatments for osteosarcopenia can prevent coronary atherosclerosis and cardiovascular events.

## Materials and methods

### Study subjects

This two-center, cross-sectional study was a part of the Kangbuk Samsung Health Study, in which the subjects were the participants in a medical health checkup program at the two Kangbuk Samsung Hospital Healthcare Centers, Sungkyunkwan University in Seoul and Suwon, South Korea^49^. The purpose of this medical health check program was to promote the health of employees by regular check-ups and to enhance early detection of existing diseases. The population of this study consisted of a subset of participants who underwent both cardiac CT and dual-energy x-ray absorptiometry (DEXA) as part of a health examination from 2012 to 2017 (n = 32,168). Cardiac CT was performed to estimate their CAC scores, which became a common screening tool for CVD^50^. Over 80% of participants were employees of companies and government organizations and their spouses. In South Korea, the Industrial Safety and Health Law requires biennial or annual health screening exams of all employees free of charge. Other participants were people who voluntarily underwent examinations.

**Figure 2:**
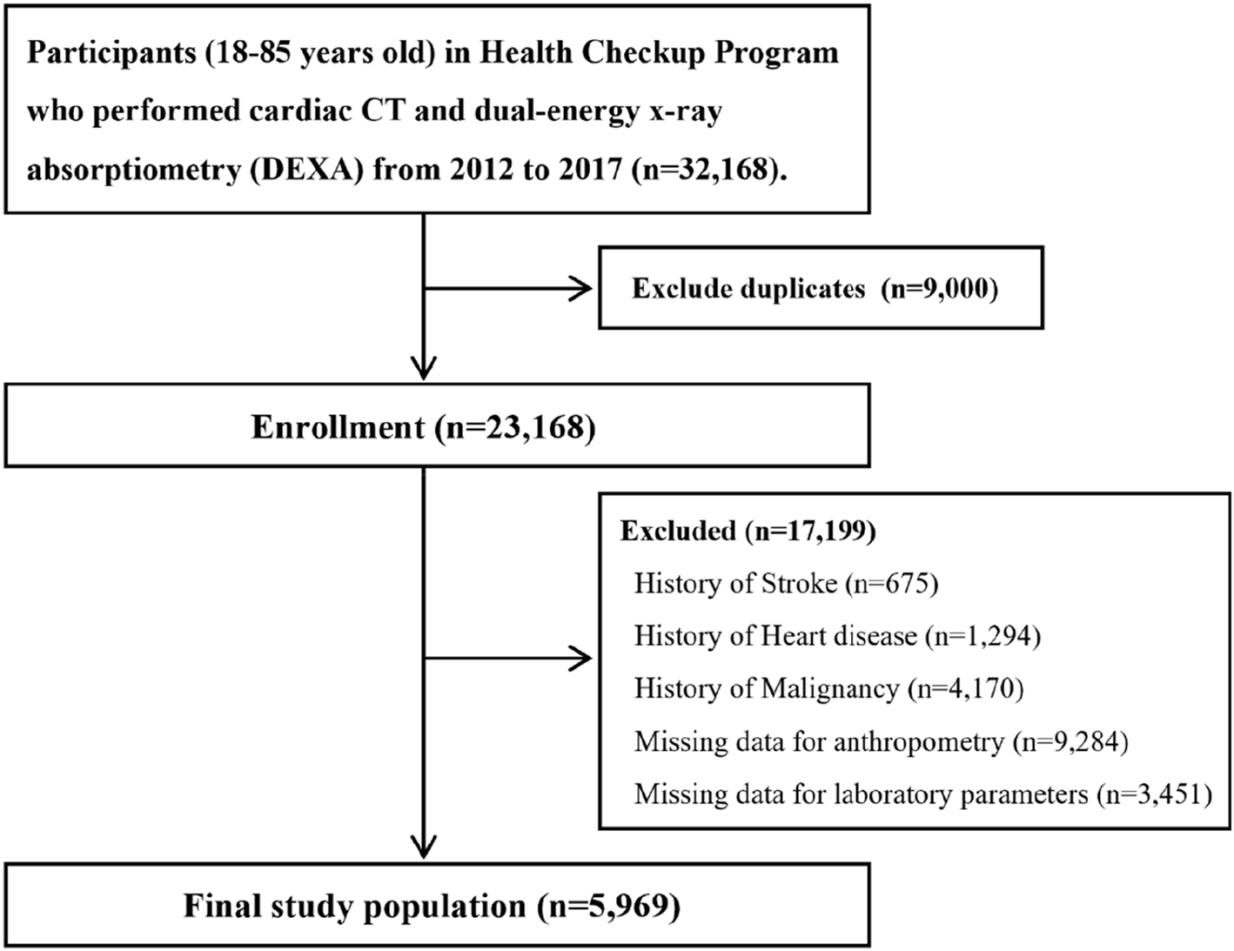
Selection of the study participants.

For this cross-sectional study, we excluded 17,199 subjects who met the following exclusion criteria: history of stroke (n = 675), history of CVD (n = 1294), history of malignancy (n = 4170), no anthropometry data (n = 9284), and missing data for laboratory parameters (n = 3451). Some participants met more than one exclusion criteria, leaving 5969 study subjects included in the final analysis (Fig. 2).

### Measurements

All participants were examined after a 12-h overnight fasting. Blood samples were collected for determining levels of triglycerides, total cholesterol, low-density lipoprotein cholesterol (LDL-C), high-density lipoprotein cholesterol (HDL-C), glycated hemoglobin (HbA1c), fasting glucose, fasting insulin, aspartate transaminase (AST), alanine aminotransferase (ALT), calcium, and phosphate. Lipid levels were measured with an enzymatic colorimetric assay. The hexokinase method was used to measure blood glucose levels. An enzymatic calorimetric test was used to measure triglycerides and total cholesterol levels. The selective inhibition method was used to measure HDL-C. The homogeneous enzymatic calorimetric test was used to estimate LDL-C. Fasting blood glucose (mmol/L) levels were estimated by a nephelometric assay with a BNII nephelometer (Dade Behring, Deerfield, IL, USA). HbA1c was calculated using an immune-turbidimetric assay with a Cobra Integra 800 analyzer (Roche Diagnostics, Switzerland). Insulin resistance was calculated using the following formula: Homeostatic Model Assessment for Insulin Resistance (HOMA-IR) = fasting insulin (μU/mL) × fasting glucose (mg/dL)/405. Serum CRP levels were measured by nephelometric assay using a BNII nephelometer (Dade Behring, Deerfield, IL, USA).

Data on smoking status, alcohol history, and medical history of CVD, stroke, hypertension (HTN), diabetes mellitus (DM), and hyperlipidemia were collected by the examining physicians using standardized, self-administered questionnaires^51^. History of CVD was defined as participants who reported physician-diagnosed CVD including myocardial infarction, angina, heart failure, and stroke (hemorrhagic or ischemic). Participants with alcohol consumption over 20 g/day were grouped into a heavy drinking group. Blood pressure was measured using a standardized sphygmomanometer after resting for 5 min according to the Hypertension Detection and Follow-up Program protocol. History of hypertension was defined as blood pressure ≥ 140/90 mm Hg or presently taking antihypertensive medication according to the criteria by the 8th report of the Joint National Committee on prevention, detection, evaluation, and treatment of high blood pressure. History of DM was determined using the diagnostic criteria of the American Diabetes Association and answers to the questionnaire. Participants with smoking status were categorized into never, former, and current smoking categories.

Anthropometric data were measured by experienced nurses. Each participant’s height and weight were measured twice and then averaged. Body mass index (BMI) was estimated as body weight in kilograms divided by height in meters squared (kg/m^2^). Waist circumference (WC) was measured as the smallest circumference between the lower end of the sternum (xiphoid process) and the umbilicus in a standing position. Appendicular skeletal muscle mass (kg) was estimated using a bioelectrical impedance analysis (BIA) with eight-point tactile electrodes (InBody 720, Biospace, South Korea). BIA was calibrated every morning before the test and validated for accuracy and reproducibility for estimating skeletal muscle mass.

### Measurement of calcified atherosclerotic plaque in coronary arteries with a multi-detector CT

A multi-detector computed tomography (MDCT) for CAC scoring was undertaken using a Lightspeed VCT XTe-64-slice, spiral CT scan (GE Health Care, Tokyo, Japan) in both Seoul and Suwon centers using the same standard scanning protocol of 2.5-mm in thickness, 400 ms of rotation time, 120 kV of tube voltage, and 124 mAs (310 mA × 0.4 s) of tube current using ECG-gated dose modulation. CT images were analyzed by one of three experienced radiologists who were blinded to clinical data of participants. The severity of CAC was calculated with the Agatston score^52^.

The presence of CAC was determined by CAC score > 0. CAC score ≥ 100 was defined as moderate-to-extensive CAC representing advanced coronary calcification^15^, known to be associated with a higher risk of overt heart disease or heart attack^53^.

As values of CAC score were extremely skewed, CAC score was analyzed in natural logarithm form plus 1: log(CAC score + 1) as previously reported. Intra-observer reliability and inter-observer reliability for CAC scoring were both excellent (intraclass correlation coefficient [ICC] of 0.99)^54^.

### Definition of osteosarcopenia

To define the status of sarcopenia, appendicular skeletal muscle mass index (SMI) was calculated as the ratio of appendicular skeletal muscle mass and height square (kg/m^2^). Sarcopenia was defined according to the criteria of the Asian Working Group for Sarcopenia (AWGS) (SMI of below 5.7 kg/m^2^ in women and below 7.0 kg/m^2^ in men)^55^. Osteoporosis was defined according to the criteria of the World Health Organization (WHO): bone mineral density (BMD) 2.5 SDs or more below the young adult mean (T-score ≤ − 2.5)^56^. BMDs were measured from DXA of the lumbar spine (L2–L4) and total hip. Osteosarcopenia was defined as the presence of both osteoporosis and sarcopenia^57^.

### Statistical analysis

Study participants were grouped into four categories: control (Non-sarcopenic/non-osteoporotic), sarcopenia alone, osteoporosis alone, and osteosarcopenia. Baseline characteristics of these groups were compared using Chi-square test for categorical variables and one-way analysis of variance (ANOVA) for continuous variables. The distribution of continuous variables was evaluated, and right-skewed variables (triglycerides, CRP, and HOMA-IR) were log-transformed for ANOVA. Adjusted means were compared between study groups using analysis of covariance (ANCOVA) after adjusting for age, sex, screening center, triglycerides, BMI, history of hypertension, CRP, HOMA-IR, smoking status, and alcohol intake.

To evaluate the association between CAC and different body compositions, a binomial logistic regression model was used to estimate odds ratios (ORs) with 95% confidence intervals (CIs) for CAC as a dependent variable. Odds ratios (ORs) were calculated as risks for the presence of CAC in the sarcopenia alone group, the osteoporosis alone group, and the osteosarcopenia group compared to the control group. We used three models to progressively adjust for potential confounders. Model 1 was crude analysis without adjustments. Model 2 was adjusted for age, sex, screening center, and triglycerides. In model 3, we further adjusted for BMI, history of hypertension, CRP, HOMA-IR. In model 4, we further adjusted for smoking status, and alcohol intake. Moreover, CAC score was introduced as a continuous variable. Multivariable-adjusted coefficients (95% CI) were estimated according to multivariable general linear models using natural log (CAC + 1) as the outcome for increasing CAC score according to the presence of sarcopenia and/or osteoporosis.

We performed stratified analyses for subgroups defined by sex (men vs. women), age (< 60 years vs. ≥ 60 years), smoking status (current smoker vs. non- or ex- smoker), alcohol intake (< 20 g/day vs. ≥ 20 g/day), HOMA-IR (< 2.5 vs. ≥ 2.5), and BMI (< 23.0, non-overweight vs. ≥ 23.0, overweight). Interactions by subgroups were conducted using likelihood ratio tests comparing models with and without multiplicative interaction terms. The level of statistical significance was set at *p* < 0.05. All analyses were conducted using IBM SPSS version 23.0 (IBM Co., NY, USA).

### Ethics approval

Ethics approval for the study protocol and data analysis was obtained from the Institutional Review Board (IRB) of Kangbuk Samsung Hospital (IRB No. 2020-08-024). This study was conducted in accordance with the 1975 Declaration of Helsinki. Informed consent was waived by the Institutional Review Board of Seoul National University Hospital because the researchers retrospectively assessed de-identified data for analytical purposes.

## Data Availability

The datasets analyzed for the study are available from the corresponding author on reasonable request.
